# Extending ImmunoSpot^®^ Assays’ Sensitivity for Detecting Rare Antigen-Specific B Cells to One in a Million—And Possibly Lower

**DOI:** 10.3390/vaccines14010088

**Published:** 2026-01-15

**Authors:** Greg A. Kirchenbaum, Noémi Becza, Lingling Yao, Alexey Y. Karulin, Paul V. Lehmann

**Affiliations:** Research and Development, Cellular Technology Ltd. (CTL), Shaker Heights, OH 44122, USA; noemi.becza@immunospot.com (N.B.); lingling.yao@immunospot.com (L.Y.); alexey.karulin@immunospot.com (A.Y.K.)

**Keywords:** ELISPOT, FluoroSpot, immunological memory, cross-reactivity, Ig class, diagnostics, assay validation, vaccine

## Abstract

**Background/Objectives**: Despite clonal expansion during a primary immune response, or after subsequent antigen encounters, the frequency of memory B cells (B_mem_) specific for an antigen remains low, making their detection difficult. However, unlike serum antibodies, which have a short half-life in vivo and thus require continuous replenishment to maintain stable titers, circulating B_mem_ are long-lived; they preserve immunological preparedness through their ability to rapidly engage in recall responses and differentiate into antibody-secreting cells (ASCs) upon antigen encounter. To this end, development of assays suited for the reliable detection of rare antigen-specific B_mem_ is critical and can provide insights into an individual’s antigen exposure history and immune status beyond that offered by traditional serum antibody measurements alone. **Methods**: ImmunoSpot^®^ has emerged as a suitable technique for the detection of individual antigen-specific B cells through visualizing their antibody-derived secretory footprints. Here, we report the theoretical and practical foundations for detecting rare antigen-specific B_mem_ in human peripheral blood mononuclear cells (PBMC). Leveraging the unique availability of verifiably naïve vs. antigen-experienced human samples, we used SARS-CoV-2 Spike (S-) and Nucleocapsid (NCAP) antigens to interrogate the presence of B_mem_ with these respective specificities. **Results**: While 100% diagnostic accuracy was achieved for both antigens, detection of NCAP-specific B_mem_ required reducing the lower detection limit of the standard assay. Specifically, this was achieved by testing a total of 2 million PBMC across multiple replicate assay wells and assessing the cumulative number of secretory footprints detected. **Conclusion**: The protocols described here should facilitate the reliable detection of ASCs present at varying precursor frequencies and serve as guidance for routine immune monitoring of rare B_mem_ with specificity for any antigen.

## 1. Introduction

Techniques for detection of antibodies in bodily fluids are well-established, and because these are very robust molecules after removal from the body they can easily be stored or shipped as serum samples. Their detection and characterization have thus become the “gold standard” for immune monitoring [1,2,3,4,5,6,7,8,9]. The presence of antibodies is the common criterion currently used for diagnosing previous antigen exposures or infections, and for predicting the magnitude (antibody titer) and quality (antibody class and subclass) of the immunity that exists in a given human for the antigen of interest. Recent observations, however, call the validity of such a solely antibody-based immune diagnostic approach into question. For example, serum antibody titers specific for SARS-CoV-2 antigens can rapidly wane after infection or vaccination [10,11,12,13,14,15,16,17,18,19], and false negative sero-diagnostic results can occur shortly after verified infections [20,21,22,23,24,25] even in the continued presence of detectable antigen-specific B_mem_ [21,26,27].

There are at least three important reasons why B_mem_ promise to become more reliable indicators of past antigen exposures than serum antibody reactivity. First, in vivo, antibody molecules are relatively short lived: IgG, for example, has a half-life of three weeks [28,29]. Therefore, the continued presence of serum antibodies requires their ongoing replenishment by plasma cells (PC). Second, while PC can potentially be long-lived, their survival depends on competition for residential niches in the bone marrow [30,31] whereas B_mem_ are long-lived and their survival does not have such limitations [32,33,34]. Third, during the immune response, the differentiation of proliferating B cells into PC and B_mem_ relies on different instructive signals [35,36]. As a result, B_mem_ and PC are not necessarily generated at similar ratios and B_mem_ can outnumber PC. Consequently, it is not uncommon to identify individuals with low or negative antibody titers (reflecting PC status) while simultaneously possessing elevated frequencies of antigen-specific B_mem_ [21,26,37].

As well as providing improved accuracy regarding antigen exposure histories, there are several additional reasons why immune monitoring of B_mem_ is of critical importance for immune diagnostics [38]. As stated above, antibodies provide only a fading image of past exposures. In contrast, B_mem_ constitute the cellular basis of future immune responsiveness. Not only do their frequencies predict the magnitude of future B cell responses, their Ig class/subclass expression also permits insights into the quality thereof. Furthermore, the distribution of BCR affinities in the antigen-specific B_mem_ repertoire [39] and the cross-reactivity of individual B_mem_ clones with future antigenic variants [40] also provides additional prognostic information. It is therefore of crucial importance to overcome the technical challenges posed by the scarcity of antigen-specific B_mem_ and the fragility of the viable cellular material required for their detection.

The use of fluorescently labeled antigen probe(s) to stain antigen-specific B cells for subsequent flow cytometry (FC) analysis is one emerging approach for B_mem_ detection, and ELISPOT/FluoroSpot (collectively called ImmunoSpot) is another [41,42]. The latter technique not only allows detection at the single cell level, but also assesses functionality by measuring antibody-derived secretory footprints originating from individual antigen-specific B cells (see Supplementary Figure S1). A recent side-by-side comparison of FC vs. ImmunoSpot documented that both techniques are equally well-suited for the detection of SARS-CoV-2 Spike (S-antigen)-specific B_mem_ and offered equivalent (100%) diagnostic accuracy—provided that the frequency of antigen-specific B_mem_ was in the high to intermediate range [43]. However, owing to the inherent background noise associated with the antigen probe-based FC approach [41,44], and the substantially greater amounts of PBMC required relative to ImmunoSpot, we hypothesized that the ImmunoSpot methodology should be best suited for measurements of low to very low B_mem_ frequencies. Here we report our successful extension of the lower detection limit of ImmunoSpot assays from the >1 in 10^5^ to the <10^6^ frequency range.

Even endemic virus-specific B_mem_ frequencies are commonly present only in very low numbers in PBMC, including those for HCMV, EBV and seasonal influenza [45]. While it can safely be assumed that essentially all individuals have been previously exposed to such viruses, when such past infections occurred typically cannot be ascertained. This is in stark contrast to exposure to the SARS-CoV-2 virus. Before the emergence of this virus in 2019 [46,47], all humans were verifiably immunologically naïve—hence their PBMC serve as perfect negative controls for detecting B_mem_ specific for the SARS-CoV-2 S-antigen and Nucleocapsid (NCAP) proteins. Conversely, PBMC obtained from individuals that had recently (within weeks to a few months) recovered from PCR-verified SARS-CoV-2 infection serve as ideal positive controls. Notably, elevated frequencies of S-antigen-specific B_mem_ were readily detected in nearly all convalescent donors ~30 days after symptom onset using fluorescently labeled S-antigen detection probes and remained elevated in longitudinally tracked donors [37]. However, frequencies of NCAP-specific B_mem_ were considerably lower in the same cohort, and a greater number of individuals’ PBMC samples yielded results below the assay’s limit of detection when tested under standard FC assay conditions. Reliably detecting NCAP specific B_mem_ is critical for understanding the spread of SARS-CoV-2 infection nowadays because the diagnostic utility of detecting S-antigen-specific serum IgG or B_mem_ for evidencing past SARS-CoV-2 infection has been lost due to widespread COVID-19 mRNA S-antigen vaccination [48].

To this end, and in line with our ongoing efforts to extend the detection limit for rare antigen-specific B_mem_, here we focus on NCAP-specific B_mem_. To achieve our ambitious goal of reliably detecting B_mem_ that are present in the ultra-low frequency range, we specifically needed to establish the scientific basis and best practices for quantifying very rare antigen-specific B_mem_ using the ImmunoSpot methodology. Importantly, the lessons learned within the unique highly controlled SARS-CoV-2 model are also directly applicable to the detection of B_mem_ with specificity for other antigens, especially in instances where the individual’s prior exposure history cannot be verified.

## 2. Materials and Methods

### 2.1. Human Subjects

Peripheral blood mononuclear cells (PBMC) tested in this study originate from the ePBMC^®^ library (Cellular Technology Limited (CTL), Shaker Heights, OH, USA). These samples were collected at FDA-registered centers and were obtained from IRB-consented healthy human donors by leukapheresis and were then sold to CTL, identifying donors only by code and concealing the subjects’ identities. PBMC were isolated and cryopreserved according to previously described protocols [49]. All PBMC samples were stored in liquid nitrogen until testing. Details of all human donors included in this manuscript are provided in Supplementary Table S1.

### 2.2. Murine B Cell Hybridoma

A murine B cell hybridoma line (M-F10-D8) secreting an IgG1, κ monoclonal antibody (mAb) with reactivity against the SARS-CoV-2 Spike protein (S-antigen) expressed by the Wuhan-Hu-1 strain was generously provided by Dr. Giuseppe Sautto (Cleveland Clinic, Port St. Lucie, FL, USA). In brief, the M-F10-D8 B cell hybridoma line was generated from a DBA/2J mouse (Jackson Laboratory, Bar Harbor, ME, USA) immunized intraperitoneally with heat-inactivated SARS-CoV-2 virus antigen [50] (~100 µL of virus antigen) adjuvanted with aluminum hydroxide on day 0, day 21, and day 42. To further focus the B cell response towards the S-antigen, the mouse was boosted intraperitoneally with 15 μg of SARS-CoV-2 Spike (S-antigen) protein [51] on day 63. Following an intraperitoneal booster immunization with 15 µg of S-antigen in PBS on day 77, fusion of the splenocytes with SP2/0 myeloma cells was performed four days later, following similar methods as described previously [52]. The M-F10-D8 B cell hybridoma was single-cell cloned by FACS prior to its shipment, and was passaged in complete medium consisting of RPMI 1640 (Alkali Scientific, Fort Lauderdale, FL, USA) supplemented with 10% fetal bovine serum (Gemini Bioproducts, West Sacramento, CA, USA), 100 U/mL penicillin, 100 U/mL streptomycin, 2 mM L-Glutamine, 1 mM sodium pyruvate, 8 mM HEPES (all from Life Technologies, Grand Island, NY, USA), and 50 µM β-mercaptoethanol (Sigma-Aldrich, St. Louis, MO, USA) at CTL until achieving a viability of >90% before use in assays.

### 2.3. Recombinant Proteins

Recombinant SARS-CoV-2 Spike (S-antigen) protein representing the ancestral Wuhan-Hu-1 strain [51] was acquired from the Center for Vaccines and Immunology (CVI) (University of Georgia (UGA), Athens, GA, USA). Recombinant hemagglutinin (rHA) proteins encoding A/California/04/2009 (CA/09, H1N1), A/Texas/50/2012 (TX/12, H3N2) and B/Phuket/3073/2013 (Phuket/13, B/Yam) were also obtained from the CVI. Recombinant Epstein–Barr virus nuclear antigen 1 (EBNA1) protein was purchased from Serion (Würzburg, Germany). The human cytomegalovirus (HCMV) gH pentamer complex, consisting of gH, gL, UL128, IL130 and UL131A proteins, was purchased from The Native Antigen Company (Kidlington, UK). Recombinant SARS-CoV-2 Nucleocapsid (NCAP) protein was purchased from the Wuhu Interferon Biological Products Industry Research Institute (Wuhu, China). Importantly, all recombinant proteins used in this study possessed a genetically encoded (His) affinity tag.

### 2.4. B Cell ImmunoSpot^®^ Assays

For detection of antigen-specific ASCs using the affinity capture coating method [53], PVDF membrane bottomed 96 well ImmunoSpot^®^ assay plates (CTL) were first pre-conditioned with 70% (*v*/*v*) EtOH (15 μL/well) followed by two washes with phosphate-buffered saline (PBS) (150 μL/well) prior to coating with purified anti-His antibody at 10 µg/mL in Diluent A (provided in CTL’s affinity coating kits) overnight at 4 °C. The following day, assay plates were washed once with 150 μL/well of PBS and then coated overnight at 4 °C with His-tag labeled recombinant SARS-CoV-2 S-antigen or NCAP at 10 µg/mL in Diluent A. Prior to use, assay plates were washed once with 150 μL/well of PBS and then blocked with 150 μL/well of complete medium for 1 h at room temperature. Immediately prior to plating cells, assay plates were decanted, and 100 μL/well of pre-warmed complete medium was added to each well.

PBMC were harvested after 5 days of polyclonal stimulation [40,49] and washed twice with PBS prior to counting using CTL’s Live/Dead Cell Counting Suite on an S6 Flex M2 Analyzer (CTL). After centrifugation, cell pellets were resuspended at 5 × 10^6^ live cells/mL in complete medium, or were diluted to 2 × 10^6^ live cells/mL, prior to plating into assays unless otherwise indicated. For S-antigen assays, donor PBMC were tested using a two-fold serial dilution approach starting at 2 × 10^5^ live cells per well unless otherwise indicated. To avoid damage to the assay membrane, PBMC were serially diluted in round-bottom 96-well tissue culture plates (Corning, Sigma-Aldrich) and then subsequently transferred into assay plates as previously described [49]. For NCAP assays, donor PBMC were tested in four replicate wells input with either 2 × 10^5^ or 5 × 10^5^ live cells per well unless otherwise indicated. Following plating of PBMC, assay plates were incubated for 4–6 h at 37 °C, 5% CO_2_. In previous studies we established that this incubation time is both needed and optimal for revealing the secretory footprints of the individual ASC using the ELISPOT methodology; before diffusion of the released antibodies into the supernatant causes an elevation of background membrane staining via an “ELISA effect” [54]. Plate-bound spot-forming units (SFUs), each representing the secretory footprint of a single ASC, were visualized using IgG-specific detection reagents included in the human IgG Single-Color Enzymatic ImmunoSpot^®^ kit (from CTL) according to the manufacturer’s instructions. Likewise, Human or murine pan IgG ELISPOT assays were performed using reagents included in Single-Color Enzymatic ImmunoSpot^®^ kits according to the manufacturer’s instructions. It is worth noting that IgG-secreting ASCs (representing recently differentiated plasmablasts that are in transit from lymphoid tissues to the bone marrow) may be present in PBMC acutely after an antigen encounter and such ASCs can be detected by plating PBMC directly into the assay, without prior polyclonal stimulation [55,56,57,58,59]. All human data reported here were obtained following polyclonal stimulation of PBMC as required for the conversion of resting B_mem_ into ASCs [54]. Lastly, the anti-IgG detection reagent provided in the human IgG Single-Color Enzymatic ImmunoSpot^®^ kit does not exhibit detectable reactivity with either the anti-His or anti-Igκ/λ capture antibody reagents.

### 2.5. Image Acquisition and SFU Counting

Plates were air-dried prior to scanning on an S6 Ultimate Analyzer (CTL). SFUs were then enumerated using ImmunoSpot^®^ Single-color Studio software (Version 1.7.35.1) and the B cell ELISPOT-specific IntelliCount™ algorithm for SFU detection [60]. Assay-specific minimal size and intensity thresholds were then applied for more precise enumeration of pan, S-antigen, or NCAP-specific SFUs. Notably, to reduce counting of artifacts that were more prevalent in NCAP-coated wells input with 5 × 10^5^ PBMC, the counting parameters deployed for enumeration of NCAP-specific SFUs were established for optimal performance in wells input with 2 × 10^5^ PBMC (discussed in greater detail in Section 3.4 and Section 3.5). Only SFU counts within the linear range of pan or S-antigen-specific assays, or SFU counts from the highest cell input tested, were considered for frequency calculations and were used to extrapolate SFU counts to a defined fixed input (denoted in the corresponding figure or tables) using the B cell export template. Alternatively, the mean ± SD or cumulative sum of replicate wells tested with a fixed cell input were used to approximate the frequency of NCAP-reactive IgG^+^ ASC. Since ImmunoSpot^®^ B cell kits, analyzers, and software proprietary to CTL were used in this study, we refer to the collective methodology as ImmunoSpot^®^.

### 2.6. Statistical Methods

Correlation of determination values, denoted as *R*^2^, for frequency measurements based on three or more cell inputs from the cell dilution series were calculated using the ImmunoSpot^®^ Single-color Studio software (Version 1.7.35.1). The precision of measurements between replicate wells, or coefficient of variation (CV), was calculated by dividing the standard deviation ( σ) by the mean ($\bar{x}$) and is reported as a percentage ($\%\;CV=100\times (\sigma /\bar{x}$)). Significant differences in the frequency of pan IgG^+^ ASC in the donor cohorts detailed in Table 1 was assessed using an unpaired Student’s *t*-test (GraphPad Prism 10 Version 10.4.0, San Diego, CA, USA).

To assess whether the observed spot counts in replicate wells conformed to a Poisson distribution at low cell inputs, two independent approaches were applied: (i) quantile–quantile (Q–Q) plot analysis comparing observed count quantiles with theoretical Poisson quantiles, and (ii) a chi-squared goodness of fit test for the Poisson model. Both analyses were conducted using SpotStat™ software (CTL, Version 1.7.0.0). The absence of overdispersion in the spot-count data at low cell inputs provided additional evidence supporting Poisson distribution.

## 3. Results and Discussion

### 3.1. Rationale

In our efforts to reduce the lower detection limit for rare antigen-specific B_mem_ in PBMC we relied on the ImmunoSpot^®^ technology in which the IgG-derived secretory footprints originating from individual B cells are visualized. Based on this assay’s basic principle (illustrated in Supplementary Figure S1), there should be no inherent lower limit of detection provided that the assay has been previously optimized for measuring antibody secretion by individual antigen-specific B cells among all cells seeded into the assay. To establish the test conditions where this requirement applies was one primary aim of the work presented in this article. We elected to focus on IgG-secreting antigen-specific B cells for the tracking of PBMC-derived B_mem_ because IgG is exclusively secreted by B cells that have previously undergone in vivo antigen-driven T helper cell-dependent Ig class switching (reviewed in [36,61]) and, by this most stringent of criteria, qualify as B_mem_. Thus, antigen-specific IgG secretion in PBMC enables the identification of B cells previously generated during the course of an immune response in vivo. Importantly, since resting B_mem_ do not spontaneously secrete antibodies, to become detectable in ImmunoSpot assays they must first be polyclonally stimulated in vitro to drive their terminal differentiation into antibody-secreting cells (ASCs) [62,63].

In order to accurately detect B_mem_ at single cell resolution among PBMC in ImmunoSpot assays, we first needed to verify that IgG secretion by polyclonally stimulated primary B cells is fully autonomous, i.e., it follows the rules of a first-order reaction measuring cellular functions that are independent of other assay variables, such as the density and activity of other ASCs or third-party cells present in the culture. As this information, to the best of our knowledge, is not available in the literature for B cell ImmunoSpot assays, but is critical for detecting rare B_mem_ at single cell resolution, in the first part of this work we sought to establish conditions under which this is the case. We argue that if IgG production by polyclonally stimulated primary B cell blasts is truly autonomous during the assay’s duration, one would expect that (a) the number of secretory footprints produced by such ASCs would be linearly related to the number of cells seeded into the assay well, (b) the amount of antibody secreted by individual cells would be independent of the cell numbers seeded per well, and (c) that the detection of low frequency ASCs would follow the Poisson distribution for rare events, i.e., the inter-well variability for replicates (the sampling error) would increase profoundly as the frequency of ASCs plated per well declines. Lastly, (d) the formal proof needed to be established that the empirically measured distribution of secretory footprints matches the mathematical prediction for a Poisson distribution. If IgG^+^ ASC measurements follow such rules, their frequency can be precisely determined within PBMC, and it should be possible to reliably detect antigen-specific B_mem_ in the low and very low frequency range.

### 3.2. Studies of IgG^+^ B Cell Hybridomas in ImmunoSpot Assays

When studying primary antigen-specific IgG^+^ B_mem_ in PBMC, the true frequency of these cells is unknown and many inherent assay variables could potentially impact the results (e.g., in addition to possible antibody feedback regulation, the influence of the third-party cells that are present at different densities could affect the B cell’s productivity or the detection of individual ASCs). Therefore, we started out with the systematic analysis of a defined, biologically simple test system—autonomously IgG-secreting B cell hybridoma clones. Of several murine B cell hybridoma lines tested, results generated by an S-antigen-specific hybridoma (M-F10-D8) are presented in the following. Notably, these data are fully representative of results obtained using several other murine B cell hybridoma clones as well.

**Figure 1:**
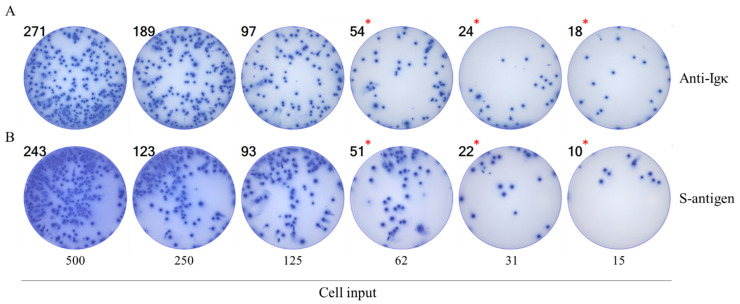
Serial dilution of B cell hybridoma cells corresponds to a reduction in detectable secretory footprints. (**A**) S-antigen-specific mAb (IgG1, κ)-producing B cell hybridoma cells (M-F10-D8) were seeded at 500 cells/well and serially diluted two-fold as indicated. IgG1-derived secretory footprints were measured in a pan (total) IgG1 assay as illustrated in Supplementary Figure S1A. The number of secretory footprints (spot-forming units, SFUs) detected was established by machine-assisted counting using ImmunoSpot^®^ software (refer to Section 2.5), with the SFU counts specified in the top left corner of the corresponding well images. Counts in which the number of SFUs detected and hybridoma cells plated per well followed a linear function are denoted with red asterisks. Note the slight increase in background membrane staining and crowding of SFUs that occurred at 250 cells/well, and to a greater extent at the 500 cells/well input resulting in undercounting relative to the expected linear outcome. (**B**) S-antigen-specific assay. The above description of the pan IgG1 assay applies, except the membrane was directly coated with S-antigen. One of four replicate wells is shown for each condition. The graphical evaluation of these results is shown in Figure 2A,B.

**Figure 2:**
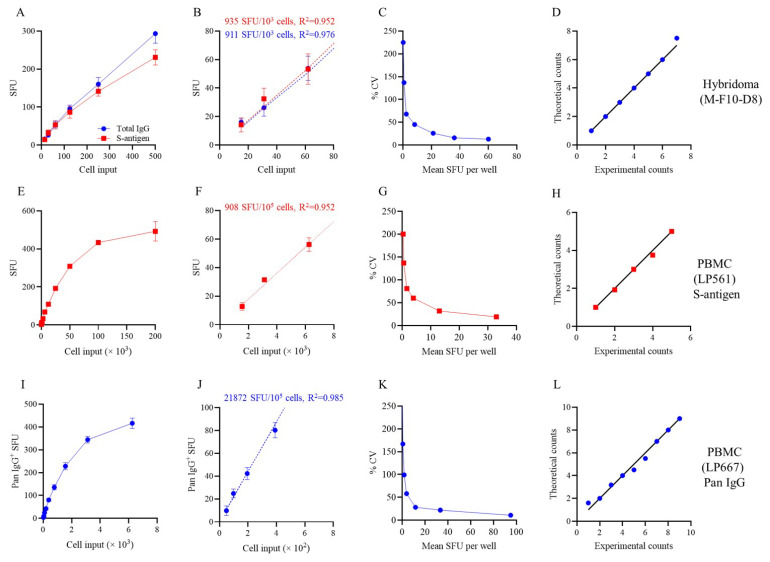
Rules for the basic evaluation of ImmunoSpot data. (**A**–**D**) Results are shown for a B cell hybridoma (M-F10-D8) line: (**A**,**B**) Graphing of spot-forming units (SFUs) described in Figure 1 with the pan IgG results denoted with blue symbols and the S-antigen-specific assay results with red symbols. Mean ± SD of four replicate wells tested for each condition are shown. Note in panel (**B**) the linearity of SFU counts and the number of hybridoma cells plated per well up to 60 SFU/well, and the flattening of the curve at higher SFU counts seen in panel A resulting from the ELISA effect and SFU crowding observable in Figure 1. The extrapolated frequency (panel (**B**)) of pan or S-antigen-specific ASC per 10^3^ cells plated, along with the coefficient of determination (*R*^2^) of the respective frequency calculations, are denoted in the inset (refer to Section 2.6). (**C**) Increasing coefficient of variation (% CV) among replicate wells at SFU counts < 10/well. M-F10-D8 hybridoma cells were plated at decreasing numbers in 48 replicate wells from which the specified % CV was calculated. Note the profound increase in % CV at low SFU counts that is consistent with a Poisson distribution for low frequency events. (**D**) Q–Q plot establishing that a Poisson distribution applies to the observed inter-well variation in M-F10-D8-derived secretory footprints with a mean = 3 SFU/well and SD = 1.7 SFU/well. Chi-square goodness of fit test for a continuous probability distribution yielded a *p*-value = 0.55 and further supports that the observed data fit a Poisson distribution model (refer to Section 2.6). (**E**–**H**) Results obtained testing for S-antigen-specific B_mem_-derived IgG^+^ ASC following polyclonal stimulation of PBMC isolated from a convalescent donor (LP561) with PCR-verified SARS-CoV-2 infection (refer to Supplementary Table S1). The PBMC were serially diluted and input into the S-antigen-specific assay. The SFU counts vs. number of PBMC plated are shown (panel (**E**)) with the deviation from linearity clearly visible starting at >75 SFU/well; panel F focuses in on the linear range. Frequency of S-antigen-specific IgG^+^ ASC per 10^5^ PBMC plated, along with the *R^2^* value, is denoted in the inset of panel (**F**). (**G**) Increasing % CV between SFU counts detected in 48 replicate S-antigen-coated wells at decreasing inputs of PBMC from LP561 is shown. (**H**) Q-Q plot establishing that a Poisson distribution applies to the observed inter-well variation in S-antigen-specific IgG^+^ secretory footprints with a mean = 1.6 SFU/well and SD = 1.3 SFU/well. Chi-square goodness of fit test for a continuous probability distribution yielded a *p*-value = 0.24 and further supports that the observed data fit a Poisson distribution model. (**I**–**L**) Results obtained testing for pan IgG^+^ ASC following polyclonal stimulation of PBMC isolated from a donor (LP667) collected in the post-COVID era (refer to Supplementary Table S1). The PBMC were serially diluted and plated in a pan IgG-detecting assay with four replicates per cell input tested. The SFU counts vs. number of PBMC plated are shown in panel (**I**) and the linear section is shown in panel (**J**). Frequency of pan IgG^+^ ASC per 10^5^ PBMC plated, together with the *R*^2^ value, is denoted in the inset of panel (**J)**. (**K**) Increasing % CV between SFU counts detected in 48 replicate wells at decreasing inputs of PBMC from LP667 is shown. (**L**) Q-Q plot of pan IgG^+^ SFU fitting a Poisson distribution in wells with a mean = 3.8 SFU/well and SD = 2.2 SFU/well. Chi-square goodness of fit test for a continuous probability distribution yielded a *p*-value = 0.76. Collectively, these data establish that all of these Ig-detecting ImmunoSpot results have a Goldilocks range between 10 and up to ~100 SFU/well in which SFU counts are linearly related to the number of ASC present in the test sample, and from which frequencies of ASC can be calculated within all cells plated. At <10 SFU/well, however, stochastic variability inherent with Poisson distributions increasingly introduces uncertainty into the detection of rare events.

Figure 1 shows images of wells obtained after plating B cell hybridomas in a two-fold serial dilution starting with an input of ~500 hybridoma cells per well. In Figure 1A, the secretory footprints (spot-forming units, SFUs) generated by the IgG1-secreting hybridoma cells were captured on the assay membrane pre-coated with antibodies specific for the kappa light chain (Igκ); subsequently, the plate-bound hybridoma-secreted IgG secretory footprints were visualized using IgG1-specific detection reagents, as illustrated in Supplementary Figure S1A. In parallel with this pan (total) IgG-detecting ImmunoSpot^®^ assay, an S-antigen-specific test was also performed in which the assay membrane was coated with the S-antigen itself, and thus the antibodies secreted by the hybridoma cells were retained on the membrane owing to their specificity for the S-antigen (Figure 1B). Following machine-assisted automated counting of the individual SFUs in the respective wells, the resulting data are also depicted in Figure 2A–D. As seen in Figure 2B, for both pan IgG detection (blue symbols) and S-antigen-specific measurements (red symbols), the number of SFUs and the number of hybridoma cells seeded was close to perfectly linear up to ~50 SFU/well. Moreover, the ASC frequencies calculated from the respective assays also matched nearly perfectly, confirming that this S-antigen-specific B cell hybridoma line was indeed clonal and that >90% of the cells secreted the monoclonal antibody (mAb). However, at higher SFU counts the relationship between hybridoma cells plated and measured SFUs began to flatten (Figure 2A). The undercounting at higher SFUs was in part due to an elevated assay background that results from the re-capture of excess hybridoma-derived antibody present in the culture supernatant distally (“ELISA effect”) as well as the crowding of SFUs (in Figure 1 clearly visible at the 500-, and to a lesser extent also the 250-cell input). The slight discrepancy between SFU counts in the pan (total) vs. the S-antigen-specific assays at higher cell inputs can be attributed to superiority of the anti-Igκ capture antibody reagent compared to the reduced—albeit still high—affinity of the secreted mAb for the S-antigen itself that resulted in more efficient capture/retention of the secreted analyte in the pan (total) IgG1 assay. Subsequently, the ELISA effect was lower for the pan IgG1 assay, and the counting of individual SFUs somewhat more reliable over the reduced background. In general, in all similar experiments performed so far, we found that the linear function between cell input and SFU counts is tight only up to 70 (conservative for most antigens and pan Ig capture antibodies) and 150 (for select assays only) SFU/well. These are the maximal SFU counts from which accurate frequencies of ASCs within all cells plated can be calculated. The lower limit of detection will be defined by the rules of Poisson distributions, if they indeed apply to B cell ImmunoSpot results.

As hybridomas produce antibody autonomously, in the absence of bystander cells that could interfere with the assay, one would expect SFU counts to follow the predictions of Poisson for the detection of rare events. One of Poisson’s predictions is that the standard deviation (SD) among replicate wells (denoted as % CV—refer to Section 2.6) will profoundly increase when fewer and fewer cells are seeded per well. Figure 2C shows that this was indeed the case for the hybridoma cells. To further test whether the rules of a Poisson distribution apply, we also studied whether the empirically detected intra-well variability of SFU counts followed those mathematically predicted for a Poisson distribution. As seen in the Q-Q plot in Figure 2D, a close to perfect match was found.

Thus, using a biologically simple hybridoma model, in which the cells plated are tumor cells that autonomously secrete IgG, we have established that secretory footprints are generated by individual ASCs under an upper limit of quantification above which SFU crowding and ELISA effects interfere with their detection. Serial dilution of the sample is readily suited to extend this upper limit of quantification—using the serial dilution strategy the test system has no inherent upper limit of quantification. As far as the lower limit of detection is concerned, with hybridomas (in the absence of other cells that might interfere) the stochastic nature of Poisson distribution limits the precision of such single-well measurements, which was increasingly more evident with cell inputs yielding <10 SFU/well. Therefore, the hybridoma data suggest that for ASC frequency calculations SFU counts between 10 and 70 (assay and donor dependent up to 150 SFU/well) provide the “Goldilocks range” on which accurate measurements can be based. After we established the above rules for autonomously IgG-secreting hybridoma cells, we next sought to test whether they also apply to polyclonally stimulated primary IgG-secreting (B_mem_-derived) ASCs that represent only a minor fraction of cells within the PBMC plated.

### 3.3. Studies of B_mem_-Derived IgG^+^ ASCs in PBMC

**Figure 3:**
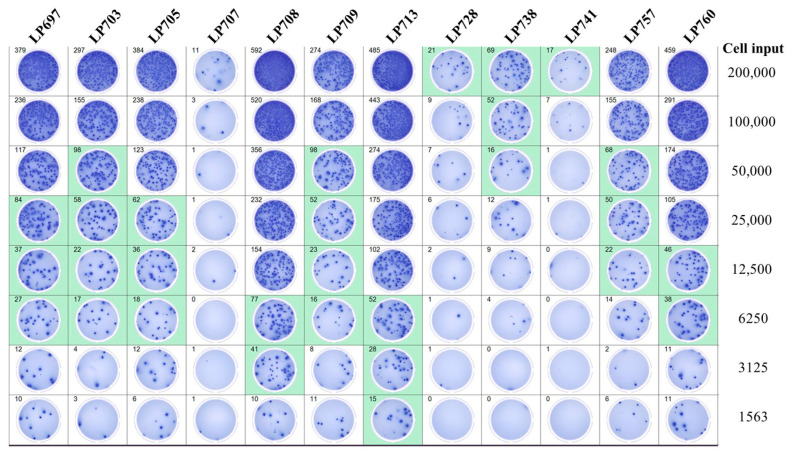
S-antigen-specific B_mem_-derived IgG^+^ ASC occur at relatively high frequencies among PBMC isolated from post-COVID era donors. A representative ELISPOT plate overview depicting testing of 12 donors collected in the post-COVID era (refer to Supplementary Table S1) for S-antigen-specific B_mem_-derived IgG^+^ ASC using a singlet serial dilution approach. The assay principle is illustrated in Supplementary Figure S1B. For each donor, the two-fold (1 + 1) serial dilution was initiated at 2 × 10^5^ PBMC/well, as specified. The raw images and machine-assisted automated SFU counts are shown in the top left corner for each well. The ImmunoSpot^®^ Studio.SC software (refer to Section 2.5) automatically denotes wells yielding SFU counts within a defined upper and lower bound with green shading; for this assay plate 15 to 100 SFU/well. Using contiguous datapoints within the so-called “Goldilocks range” the software automatically calculates the frequency of ASC within all PBMC tested; the autogenerated frequency calculations for donors yielding three or more datapoints within the defined upper and lower SFU range are shown in Supplementary Figure S2. The data highlight that frequencies of S-antigen-specific B_mem_-derived IgG^+^ ASC span a wide range in healthy individuals, requiring the serial dilution strategy for accurate calculations for most donors; however, in 3 of the 12 donors the number of SFUs detected was too low for frequency calculations using this strategy.

S-antigen-specific IgG^+^ B_mem_ are known to occur in a wide frequency range in PBMC of healthy people [45,64]. We therefore tested PBMC from random donors collected between May 2022 and May 2025 (refer to Supplementary Table S1), by which time most individuals can be expected to have either been previously infected with the SARS-CoV-2 virus and/or received one or more COVID-19 vaccine inoculations. Figure 3 shows the results of a typical S-antigen-specific IgG^+^ ImmunoSpot^®^ test with each individual’s PBMC two-fold serially diluted starting at 2 × 10^5^ PBMC/well. The resulting SFU counts, which were automatically established by machine-assisted counting, are shown in the upper left corner of each well. The software also identifies the test wells yielding SFU counts within the user-defined Goldilocks range, and denotes them with green shading. Importantly, when three or more SFU counts from a serial dilution of a subject’s PBMC fall within the Goldilocks range, these SFU counts exhibited a linear relationship with cell numbers plated. From the resulting trend line, the frequency of ASCs among all PBMC plated was extrapolated (as was performed in Supplementary Figure S2 for selected donors shown in Figure 3). Therefore, for measurements of antigen-specific IgG^+^ B_mem_ present at high frequencies in PBMC, the autonomous IgG secretion-based rules established above using B cell hybridomas also applied. However, for donors with lower frequencies of S-antigen-specific IgG^+^ B_mem_, for whom only the highest cell input (2 × 10^5^ PBMC) provided SFU counts above, or close to, the Goldilocks threshold (15 SFU/well for this assay plate), replicate wells and/or optimized assay conditions would be needed to more precisely establish accurate frequencies. This would apply to donors LP707, LP728, and LP741, 3 of the 12 donors shown in Figure 3. Related to this, we asked to what extent Poisson noise affected the detection of such low frequency antigen-specific IgG^+^ B_mem_ in ImmunoSpot^®^ tests.

As was previously seen using hybridoma cells (Figure 2A–C), testing a polyclonally stimulated PBMC sample obtained from a convalescent donor (LP561) following PCR-verified SARS-CoV-2 infection using a serial dilution approach, it was apparent that only S-antigen-specific IgG^+^ SFU counts in wells yielding between 10 and 70 SFU/well displayed a linear relationship with the number of PBMC plated (Figure 2E,F). Furthermore, S-antigen-specific SFU counts also displayed increasing well-to-well variation (denoted as % CV) when fewer and fewer PBMC were plated and, consequently, the number of S-antigen-specific IgG^+^ ASC was <10 SFU/well (Figure 2G). Lastly, we verified that the empirically measured SFU counts at low PBMC inputs also conformed to a Poisson distribution (Figure 2H).

Similar results were also obtained when measuring pan (total) IgG^+^ ASC following polyclonal stimulation of PBMC (the example shown in Figure 2I–L is from testing the PBMC of donor LP667). Supplementary Figure S3 shows the raw data supporting what was already shown for S-antigen-specific B_mem_-derived IgG^+^ ASC in Figure 3. Namely, the frequency of pan IgG^+^ ASC following polyclonal stimulation of donor PBMC also showed considerable inter-donor variability and necessitated a serial dilution approach. In other words, there is no single cell input at which SFU counts within the Goldilocks range can be reliably assumed for all individuals’ samples. Owing to ELISA effects and crowding, SFU counts were also underestimated in the pan IgG assay when they exceeded 150 SFU/well; but, between 10 and 150 SFU/well, the SFU counts and PBMC input were linearly related and permitted frequency calculations (Figure S4 and Figure 2J). However, when SFU counts were <10 SFU/well, increasingly the influence of noise resulting from Poisson distributions prevailed in pan IgG^+^ ASC measurements as well (Figure 2K,L). Having thus established the basic rules for ImmunoSpot-based assessment of IgG^+^ ASC that are relatively abundant in PBMC, we next sought to achieve reliable detection of B_mem_ existing in the low frequency range among PBMC. The Poisson rule permits us to define the number of replicate wells needed to establish precise frequencies for very rare B_mem_ (see Supplementary Figure S5).

### 3.4. Improving the Detection Limit of Rare Antigen-Specific IgG^+^ B_mem_ in ImmunoSpot Assays

**Figure 4:**
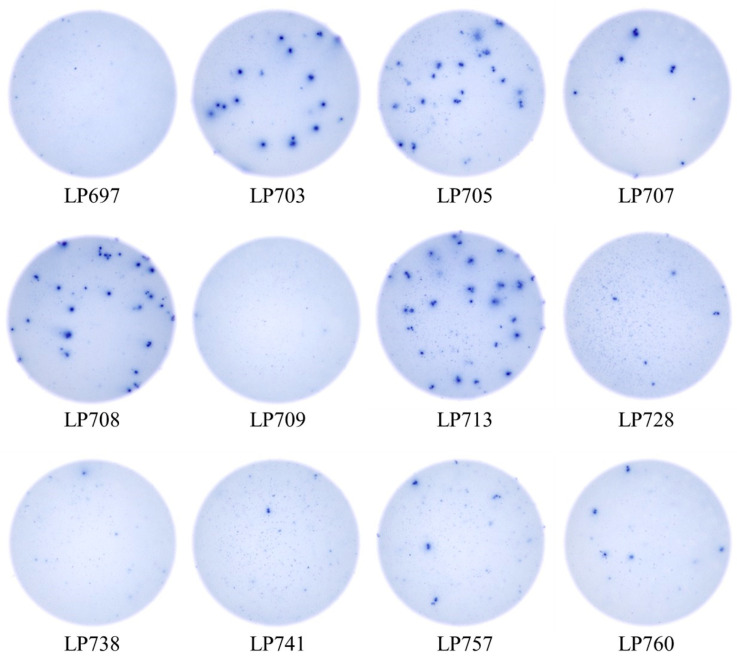
SARS-CoV-2 Nucleocapsid (NCAP)-specific B_mem_-derived IgG^+^ ASC occur at low frequencies in PBMC isolated from post-COVID era donors. Representative well images depicting test results obtained using the same post-COVID era donors shown in Figure 3 when plating 2 × 10^5^ PBMC in NCAP-coated wells. The assay principle is illustrated in Supplementary Figure S1B; refer to Section 2.5 for additional details. Note that at this PBMC plating density NCAP-specific B_mem_-derived IgG^+^ SFU counts could occur in the Goldilocks range (between 10 and 100 SFU/well, 4 donors) or were rare (<10 SFU/well, 8 donors) and therefore susceptible to variations associated with a Poisson distribution.

The data in Figure 3 established that testing for S-antigen-specific B_mem_-derived IgG^+^ ASC starting at 2 × 10^5^ PBMC per well using a serial dilution in singlet wells provided the expected positive results for all individuals whose PBMC were isolated after May 2022, although 3 of these 12 exhibited lower S-antigen-specific frequencies. When these same individuals were tested for B_mem_-derived IgG^+^ ASC reactivity for the SARS-CoV-2 Nucleocapsid (NCAP) protein using an equivalent input of 2 × 10^5^ PBMC/well, notably only one-third of them (4 of 12) yielded SFU counts that were >10 SFU/well (Figure 4). The remaining eight either yielded no detectable SFUs or generated < 10 SFU/well at the 2 × 10^5^ PBMC input. Consequently, these data did not enable a clear distinction between whether these individuals had only been previously COVID-19 vaccinated (that would reconcile their positivity for S-antigen and negative NCAP test result) or whether such donors, in addition to potentially being COVID-19 vaccinated, had previously been SARS-CoV-2 infected but did not develop elevated frequencies of NCAP-specific IgG^+^ B_mem_. Likewise, even for endemic viruses, such as seasonal influenza, EBV and HCMV, to which prior exposure and hence the formation of immune memory can be assumed to have occurred in most individuals, the frequency of B_mem_ specific for the respective antigens showed high inter-individual variability (Supplementary Table S2). Importantly, in many instances antigen-specific IgG^+^ B_mem_ occur in the very low frequency range and cannot be reliably assessed using existing testing practices. For this reason, we set out to extend the detection range of antigen-specific B_mem_ using ImmunoSpot. We focused here on the detection of SARS-CoV-2 NCAP-specific IgG^+^ B_mem_ because PBMC from truly naïve individuals are only available as negative controls for exposure to this virus owing to their cryopreservation prior to the emergence and circulation of SARS-CoV-2, constituting a pre-COVID era cohort.

**Figure 5:**
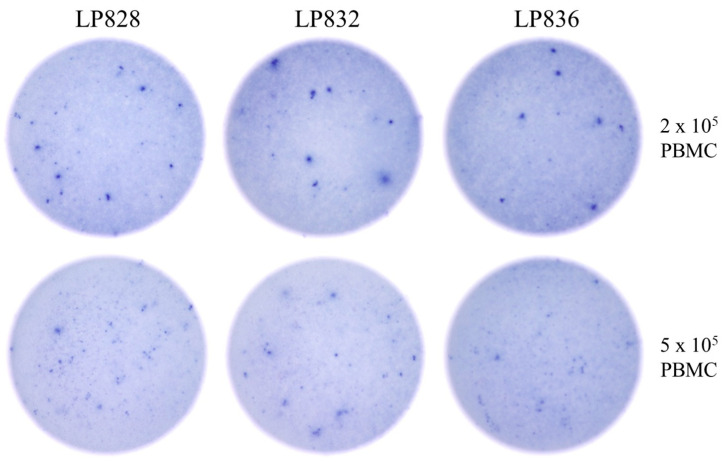
Secretory footprint morphologies generated by NCAP-specific IgG^+^ ASC deteriorate if more than 2 × 10^5^ PBMC are plated. The depicted ImmunoSpot well images were generated using PBMC from post-COVID era donors that displayed low frequencies of NCAP-specific B_mem_-derived IgG^+^ ASC in a previous test. Raw data are shown for 3 subjects out of 12 tested (see Supplementary Table S3 for summarized results) to illustrate the outcome.

Increasing the number of PBMC to be tested is the obvious solution to the detection of very rare cells. This can be accomplished in the case of ImmunoSpot assays by plating more cells per well, and/or testing many replicate wells. To test whether increasing the PBMC numbers seeded per well would improve the NCAP assay’s lower limit of detection, we selected donors whose NCAP-specific IgG^+^ SFU counts were <10 SFU when tested at 2 × 10^5^ PBMC/well. Strikingly, a higher PBMC input (5 × 10^5^) per well did not uniformly result in a proportional increase in SFU counts (Supplementary Table S3); on the contrary, in many instances the spot morphology deteriorated to a point where the secretory footprints became barely discernible (Figure 5). Moreover, the incidence of notably fainter secretory footprints and those displaying irregular morphologies also increased. Of note, to achieve objective image analysis of the corresponding well images, the counting parameters deployed were optimized for detection of secretory footprints generated in wells input with 2 × 10^5^ PBMC, which entailed applying more stringent minimal size and density criteria. We also demonstrated that this, seemingly counterintuitive, outcome is a result of third-party PBMC physically inhibiting access of ASC-secreted antibody to the membrane. The deterioration of the spot morphology was also seen when a fixed number of S-antigen-specific ASCs were tested in the presence of increasing numbers of unstimulated autologous PBMC (that, without polyclonal pre-stimulation do not secrete antibody and merely serve as third-party filler cells) (Supplementary Figure S6).

In our next attempt to extend the lower limit of detection of rare B_mem_, we increased the number of replicate wells tested. For this, a total of 2 million PBMC from select post-COVID era donors were tested across 10 replicate wells at 2 × 10^5^ PBMC/well and the cumulative number of NCAP-specific B_mem_-derived IgG^+^ SFUs was determined (Table 1). In each of the 10 post-COVID era donors tested so far, whose PBMC were isolated after May 2022, the cumulative number of SARS-CoV-2 NCAP-specific B_mem_-derived IgG^+^ SFUs was >20 SFU/2 × 10^6^ PBMC whereas the cumulative SFU count in 10 pre-COVID era donors was between 0 and 9 possible “SFUs”.

**Table 1:** Testing 2 × 10^6^ PBMC at single cell resolution permits detection of SARS-CoV-2 NCAP-specific B_mem_-derived IgG^+^ ASC with 100% diagnostic specificity in post-COVID era donors. Cryopreserved PBMC samples from ten donors collected in the pre-COVID era, and 10 in the post-COVID era (after May 2022), were polyclonally stimulated and then tested in a SARS-CoV-2 NCAP-specific ImmunoSpot assay by plating 2 × 10^5^ PBMC/well in 10 replicate wells for each donor. The cumulative number of SFUs detected in all 10 wells, i.e., a total of 2 × 10^6^ PBMC, is shown for each donor. Note, while SARS-CoV-2 NCAP “specific” SFU were seen in low numbers in select pre-COVID samples, these secretory footprints were faint and exhibited a diffuse morphology (suggesting a low-affinity/cross-reactivity) that was fundamentally different compared to the tight and dense morphologies seen when testing PCR-verified convalescent donors or post-COVID era donors. Refer to Figure 6 showing raw well images. Donor PBMC were also plated in an identical manner into 6xHis peptide- or BSA-coated wells; which served as specificity controls for comparison when testing 2 × 10^6^ PBMC. Likewise, pre-COVID era donors were also interrogated for IgG^+^ ASC reactivity for the SARS-CoV-2 S-antigen, with the cumulative SFU counts shown for each. Alternatively, frequencies of S-antigen-specific B_mem_-derived IgG^+^ ASC in the post-COVID era cohort, and pan IgG^+^ ASC activity in both cohorts, were established using a serial dilution strategy (as described in Section 2.5). SFU values were then extrapolated to 2 × 10^6^ PBMC and those determined using three or more cell inputs are denoted with an asterisk. Note, the abundance of pan IgG^+^ ASC following polyclonal stimulation did not differ between the pre-COVID era and post-COVID era donors, proving that these cells were equally functional; i.e., the lack of NCAP or S-antigen reactivity seen in the pre-COVID era donors cannot be attributed to storage-related impairment. Moreover, pre-COVID era PBMC samples yielded pristine IgG^+^ secretory footprints when plated in wells coated with hemagglutinin proteins representing seasonal influenza vaccine strains (refer to Figure 8, Figures S8 and S9).

				S-antigen ^a^	NCAP ^a^	6xHis ^a^	BSA ^a^	Pan IgG ^b^	
Pre-COVID			LP366	0	0	1	0	39,800 *	Pan IgG (µ ± σ) 133,480 ± 103,032
			LP377	3	6	3	0	157,800 *	
			LP381	0	0	0	0	19,933 *	
			LP391	0	9	0	0	57,733 *	
			LP413	3	4	0	0	299,600 *	
			LP424	1	2	0	0	49,467 *	
			LP426	0	1	2	0	257,200 *	
			LP432	2	4	1	1	70,200 *	
			LP434	0	1	3	1	133,067 *	
			LP475	0	5	4	1	250,000 *	
				**S-antigen ^b^**	**NCAP ^a^**	**6xHis ^a^**	**BSA ^a^**	**Pan IgG ^b^**	
Post-COVID			LP695	967 *	35	3	1	156,400 *	Pan IgG (µ ± σ) 115,723 ± 62,567
			LP696	5067 *	49	1	0	60,900 *	
			LP728	210	35	2	0	202,133 *	
			LP741	170	21	0	0	229,867 *	
			LP757	5200 *	31	1	0	114,133 *	
			LP815	1880 *	24	0	0	63,900 *	
			LP820	900 *	44	0	0	66,100 *	
			LP827	730 *	31	0	0	124,600 *	
			LP828	4960 *	21	0	0	52,400 *	
			LP836	1680 *	25	0	0	86,800 *	

^a^ Spot-forming units (SFUs) were aggregated from 10 replicate wells seeded with 2 × 10^5^ PBMC; ^b^ SFU values were extrapolated to 2 × 10^6^ PBMC; * SFU value determined through serial dilution of donor PBMC.

### 3.5. SFU Morphology Distinguishes Between Cognate and Cross-Reactive B_mem_

**Figure 6:**
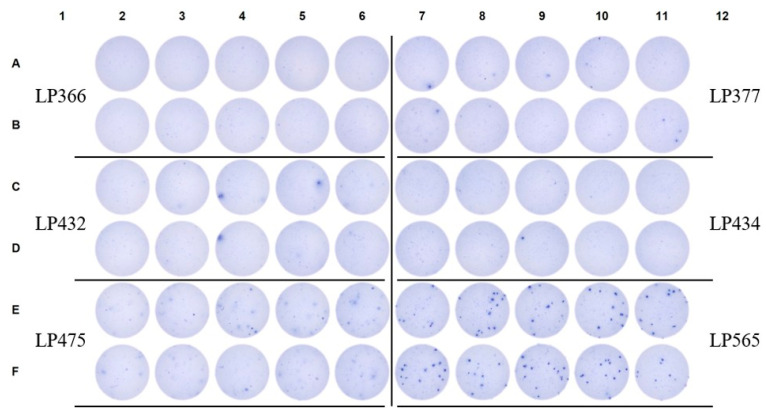
Fundamentally different SARS-CoV-2 NCAP-reactive IgG^+^ SFU morphologies are seen in pre-COVID era subjects compared to a verified SARS-CoV-2 infected donor. Raw data are shown from an NCAP-specific ImmunoSpot assay in which PBMC samples were plated into 10 replicate wells at 2 × 10^5^ cells/well. LP565 is a convalescent donor with a PCR-verified SARS-CoV-2 infection (refer to Supplementary Table S1) and served as an assay-specific positive control.

**Figure 7:**
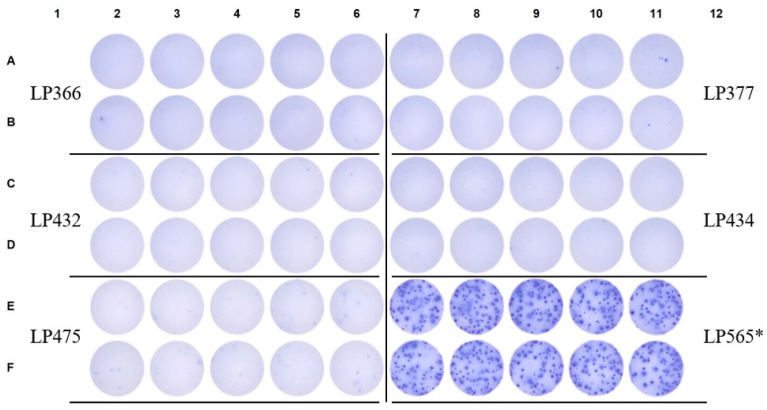
Near complete absence of S-antigen-reactive IgG^+^ SFU in pre-COVID era subjects compared to a verified SARS-CoV-2 infected donor. Raw data are shown from an S-antigen-specific ImmunoSpot assay in which pre-COVID era PBMC samples were plated into 10 replicate wells at 2 × 10^5^ cells/well. LP565 served as an assay-specific positive control and owing to their elevated frequency of S-antigen-specific B_mem_-derived IgG^+^ ASC PBMC were instead plated at 5 × 10^4^ cells/well (denoted by an asterisk).

The few SARS-CoV-2 NCAP-reactive “SFUs” that were observed when testing pre-COVID era PBMC samples exhibited a fundamentally distinct spot morphology compared to secretory footprints detected when testing a PBMC sample obtained from PCR-verified convalescent donors (in Figure 6 and Figure S7 LP565 is shown as a representative example of the latter). SARS-CoV-2 S-antigen-reactive “SFUs” were almost entirely absent in the pre-COVID cohort, and if present, these secretory footprints were also much fainter and more diffuse in their morphology compared to those generated in parallel using the positive control PBMC sample (Figure 7).

In direct antigen-specific ImmunoSpot assays, the detection of faint and diffuse SFUs is indicative of low affinity antibodies that are poorly retained on the antigen-coated membrane [54]. Therefore, the SARS-CoV-2 NCAP- (and to a much lesser extent also S antigen-) reactive secretory footprints detected when testing pre-COVID PBMC, where present, may originate from low-affinity, cross-reactive B_mem_ that were primed by prior exposure to one or more circulating common cold-causing coronaviruses (CCC), and possibly other antigens (reviewed in [65]). As the NCAP protein is more conserved than the S-antigen between SARS-CoV-2 and circulating CCC, the detection of more low-affinity SARS-CoV-2 NCAP B_mem_ in pre-COVID is, to some degree, an expected outcome. Importantly, however, the differential spot morphologies permits a clear distinction between cognate-SARS-CoV-2-antigen-primed B_mem_ and low-affinity, cross-reactive B_mem_ (refer to Supplementary Figure S7). After extending the lower limit of detection for SARS-CoV-2 NCAP-reactive B_mem_ by sampling 2 × 10^6^ PBMC per donor and also taking spot morphology into account, it was therefore possible to identify exposure to SARS-CoV-2 with 100% diagnostic specificity. Because low-affinity, cross-reactive B_mem_ can serve as precursor B cells for a cognate B cell response to SARS-CoV-2 [65,66], their presence should also have additional valuable immune diagnostic significance [38].

**Figure 8:**
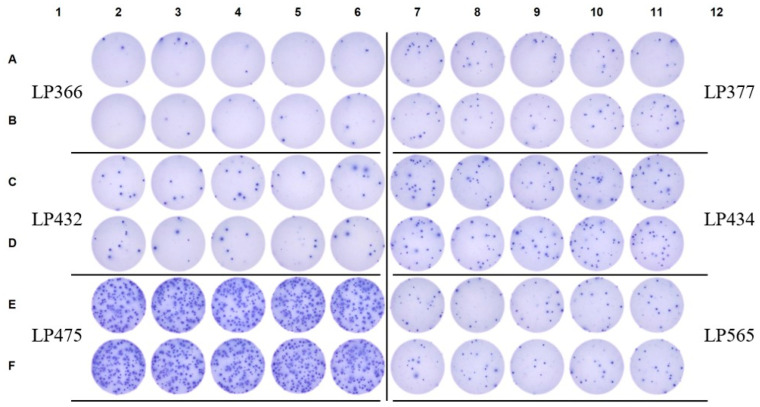
Detection of pristine influenza (H3)-specific B_mem_-derived IgG^+^ SFU in pre-COVID era donors. Raw data are shown from an ImmunoSpot assay in which pre-COVID era donors were seeded at 2 × 10^5^ PBMC/well into wells coated with recombinant hemagglutinin (rHA) protein representing a seasonal H3N2 vaccine strain (A/Texas/2012). Assay specifics were otherwise identical to those in Figure 6. Notably, while frequencies of influenza H3-specific IgG^+^ SFU were variable among the pre-COVID era donors shown, they were detectable in all subjects and were crisp and dense, i.e., reflective of high-affinity antibody binding. Moreover, these data highlight that increasing the number of replicate wells, and cumulatively the number of PBMC interrogated, in a B cell ImmunoSpot test is a universal strategy for improving the limit of detection for rare antigen-specific B_mem_—even in the absence of available negative controls.

In support of the notion that these rare and predominantly faint/diffuse SARS-CoV-2 antigen-reactive IgG^+^ SFUs detected in pre-COVID samples signify the presence of low-affinity, cross-reactive B_mem_, the same PBMC samples yielded variable numbers of influenza hemagglutinin (HA)-specific secretory footprints that, based on their morphology, likely originated from high-affinity B_mem_, i.e., the SFU were tight and dense. Raw data are shown in Figure 8 for the ImmunoSpot tests performed using a hemagglutinin (HA) representing a seasonal H3N2 vaccine strain (A/Texas/2012) for antigen coating, and in Supplementary Figures S8 and S9 using HA proteins representing an H1N1 vaccine strain (A/California/09) or an influenza B vaccine strain (B/Phuket/2013), respectively. Although for most of the donors the testing of replicate wells at 2 × 10^5^ PBMC/well was required to reliably enumerate low frequency influenza HA-specific B_mem_, the individual spot morphologies themselves were pristine and readily detected using even the most stringent machine-assisted counting parameters. Furthermore, well-to-well variation in SFU counts was also apparent when the frequency of HA-specific IgG^+^ ASC was low for a particular donor/antigen combination. As previously discussed, these observations serve as further evidence that SFU counts follow stochastic (Poisson) distributions when the frequency of antigen-specific IgG^+^ ASC is <10 SFU/well and that the testing of replicate wells is required for accurate frequency measurements.

## 4. Conclusions

Our data show that B_mem_ specific for antigens of viruses to which the human population are nowadays commonly exposed (SARS-CoV-2 included) can occur at variable frequencies in different individuals, ranging from high to very low. Hence, a single testing approach for measuring antigen-specific B_mem_ populations using the ImmunoSpot methodology, either performing a serial dilution or plating a fixed input of PBMC in many replicate wells, will rarely, if ever, be optimal for all samples. Instead, based on preliminary observations, the ImmunoSpot testing approach used for B_mem_ detection should be customized to provide the depth of resolution required. As viral infections are among the strongest immune stimuli, one can expect B_mem_ involved in less immunogenic reactions, such as self-antigen recognition during auto- or anti-tumor immunity to occur at even lower numbers. We establish here how the lower detection limit of ImmunoSpot assays can be extended to reliably detect such very rare B cells. There is, however, an upper limit of how many PBMC can be tested per well (conservatively 2 × 10^5^, or with some antigens 5 × 10^5^ PBMC/well in a standard 96 well test plate) beyond which cell crowding can impair access of the ASC-derived antibody to the membrane-absorbed antigen. When the PBMC are seeded at a maximal cell density whereby the resolution of individual ASC is still maintained, only the total number of PBMC available for testing (along with the availability of other potentially limiting reagents like the antigens themselves) defines how many replicate wells can be evaluated per donor and hence the lower limit of B_mem_ detection. This implies that ImmunoSpot assays do not have an inherent lower limit of detection.

When extending the limit of detection for rare B_mem_ using ImmunoSpot, in addition to the cumulative SFU counts revealing the number of antigen-reactive ASC within all PBMC interrogated, we further suggest that the SFU morphology should also be taken into account (Supplementary Figure S7). Namely, faint/diffuse SFUs are likely to signify the presence of low-affinity B_mem_ generated through prior exposure(s) to cross-reactive antigens. While the functional significance of low-affinity, cross-reactive IgG^+^ B_mem_ and their protective capacity in vivo is presently unclear, such class-switched B_mem_ may still constitute an appreciably increased antigen-specific precursor frequency compared to that of a truly naïve B cell repertoire [31,65]. To this end, such B_mem_, albeit not necessarily possessing an exquisitely high affinity, may still critically contribute to host immunity through their ability to rapidly differentiate into ASCs upon antigen encounter [67]. Hence, being able to monitor quantitatively (numbers) and qualitatively (affinities) the B_mem_ precursor cell repertoire (that constitutes the cellular basis for the second wall of humoral immunity) in large donor cohorts promises to open a new window for immune diagnostics that so far has largely been confined to studies of serum antibodies in retrospect. It is beyond question that the frequencies of antigen-specific B_mem_ defines the magnitude of the available precursor cell repertoire and thus the potential to mount a renewed antibody response. Therefore, identifying B_mem_ even in very low numbers signifies elevated responsiveness compared to the naïve state. How the frequencies of B_mem_ (i.e., the response potential of the host) change with repeat antigen encounters and is affected by antigen persistence is presently unknown, and gaining insights into it will largely benefit from improvements of cellular immune monitoring techniques, including high-resolution ImmunoSpot analysis. The resulting changes in B_mem_ frequencies can be expected to define host defense vs. adverse reactions and being able to measure them should add great value to monitoring an individual’s immune status vs. an antigen.

## Data Availability

The data generated in this study will be made available by the authors, without undue reservation, to any qualified researcher.

## Supplementary Materials

The following supporting information can be downloaded at: https://www.mdpi.com/article/10.3390/vaccines14010088/s1, Figure S1: Illustration of pan (total) and antigen-specific B cell ImmunoSpot^®^ test principles; Figure S2: Frequency calculations of S-antigen-specific B_mem_-derived IgG^+^ ASCs in post-COVID era donors; Figure S3: Serial dilution of donor PBMC permits measurements of pan (total) IgG^+^ ASCs; Figure S4: Frequency calculations of pan IgG^+^ ASCs in post-COVID era donors; Figure S5: Power analysis enables prediction of replicate wells required to measure low frequencies of antigen-specific ASCs with defined level of precision; Figure S6: Crowding of third-party PBMC interferes with detection of secretory footprints in ImmunoSpot assays; Figure S7: Images depicting low-affinity vs. high-affinity NCAP-reactive IgG^+^ secretory footprints at higher magnification; Figure S8: High resolution ImmunoSpot testing for influenza H1-specific B_mem_-derived IgG^+^ ASCs; Figure S9: High resolution ImmunoSpot testing for influenza B HA-specific B_mem_-derived IgG^+^ ASCs; Table S1: Donor demographics; Table S2: Viral antigen-specific B_mem_-derived IgG^+^ ASC frequencies vary considerably between individual donors; Table S3: Increasing the number of PBMC plated per well does not result in a proportional increase in NCAP-specific IgG^+^ SFU.
