# Supplementary material for: Extending ImmunoSpot® Assays’ Sensitivity for Detecting Rare Antigen-Specific B Cells to One in a Million—And Possibly Lower
Source: Vaccines (Basel). 2026 Jan 15;14(1):88. doi: 10.3390/vaccines14010088 (PMC12846458; doi:10.3390/vaccines14010088)
Supplement: Supplementary file 1 [file vaccines-14-00088-s001.zip › vaccines-4076544-Supplementary Materials.pdf]

Supplementary Materials:

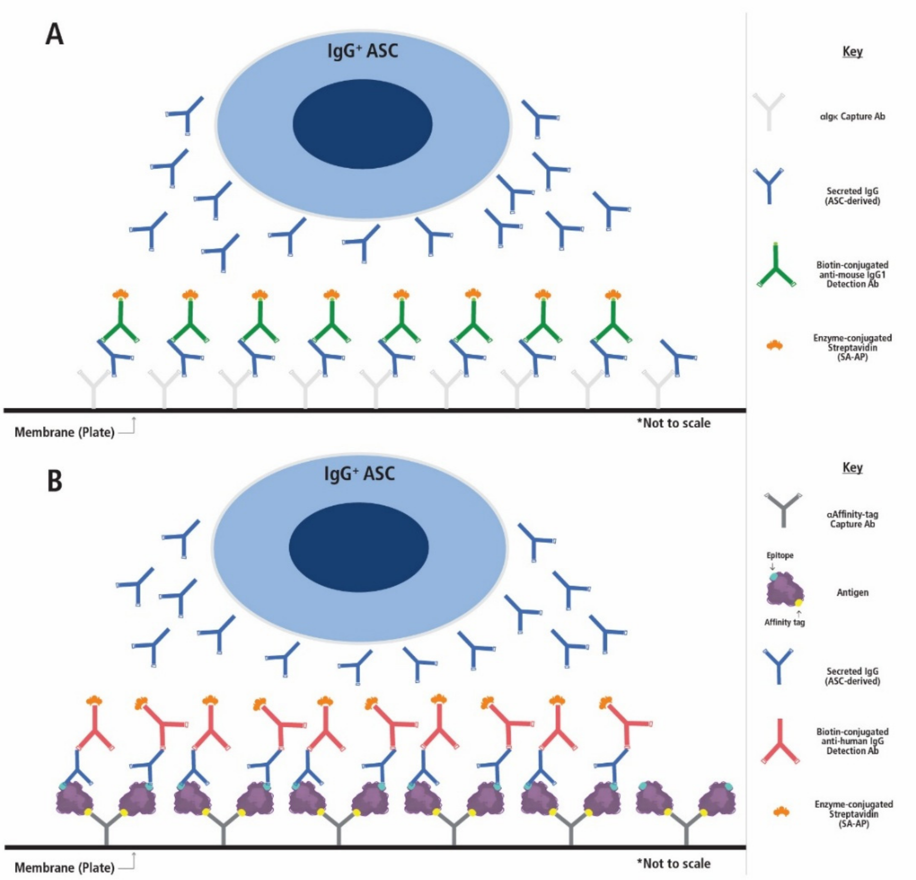

Supplementary Figure S1. Illustration of pan (total) and antigen-specific B cell ImmunoSpot® test principles. A) In a pan (total) IgG1 detection assay the Ig produced by antibody-secreting B cells (ASCs) is captured irrespective of its antigen specificity by an anti-species antibody coated onto the membrane (e.g. goat anti-mouse Igκ, depicted in gray) in close proximity to where the ASC resided on the membrane instead of being secreted into the culture supernatant. B) Alternatively, in an antigen-specific detection assay the membrane can be coated directly (not shown) or via affinity capture (as shown) with the antigen of interest to achieve high density antigen coating and maximal detection of antigen-specific ASC (31). When ASCs are plated onto such an antigen-coated surface, the antibodies produced by an antigen-specific ASC (shown in blue), as opposed to antibodies produced by ASCs specific for other antigens, will be retained on the membrane in close proximity to where the ASC resided on the membrane as a secretory footprint. In both (A) pan (mouse) IgG1 or (B) antigen-specific (human) IgG ImmunoSpot® assays, plate-bound IgG-derived secretory footprints are visualized using an anti-mouse IgG1-specific detection antibody (depicted in green) or anti-human IgG-specific (depicted in red), respectively, followed by deposition of a precipitating visible substrate in an ELISPOT (as shown). Alternatively, the detection antibody could be fluorophore-conjugated and the secretory footprints visualized through selective excitation and measurement of the resulting fluorescence using a suitable instrument (FluoroSpot, not shown).

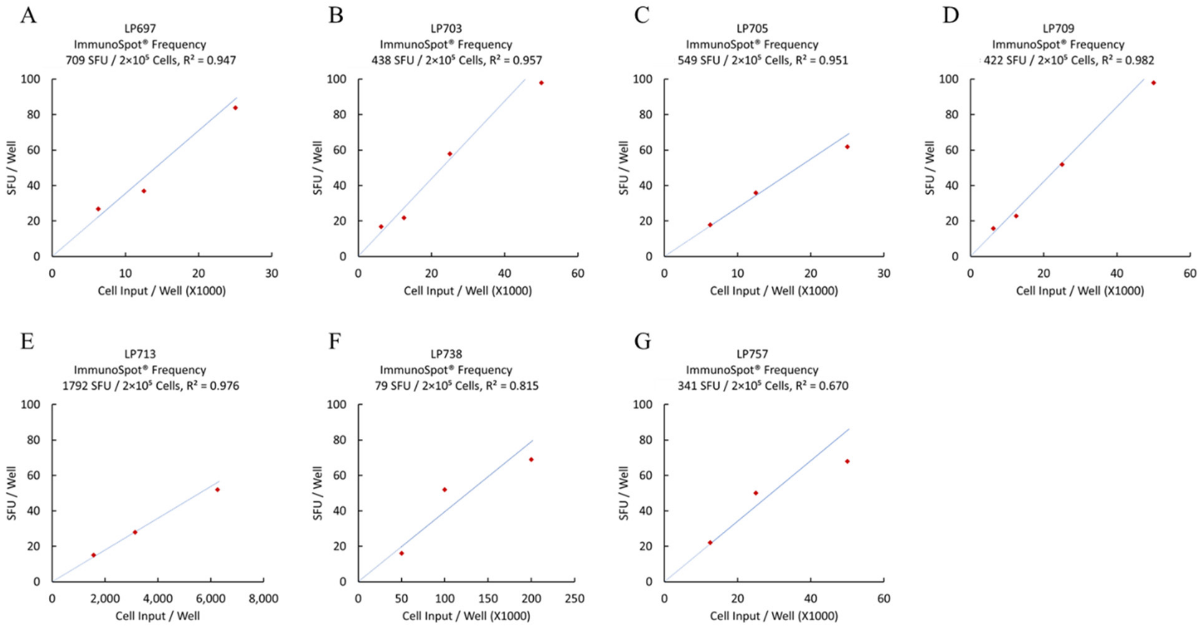

Supplementary Figure S2. Frequency calculations of S-antigen-specific B_{mem}-derived IgG^+ ASCs in post-COVID era donors. The automatically generated analysis results by the ImmunoSpot® Studio.SC software are shown for selected donors tested in the assay depicted in Figure 3 (refer to Section 2.5.). Using 3 or more datapoints within the user-defined "Goldilocks range" (between 15 and 100 SFU/well for this assay plate), the software calculates the frequency of ASC and then extrapolates SFU numbers to a desired cell input (e.g. per 2 x 10^5 PBMC). Additionally, a correlation of determination value, denoted as R^2, is also reported by the software and provides a metric for how closely the raw data at the corresponding cell inputs yielding SFU counts within the linear range fit the calculated frequency; larger R^2 values denote a higher confidence in the reported frequency.

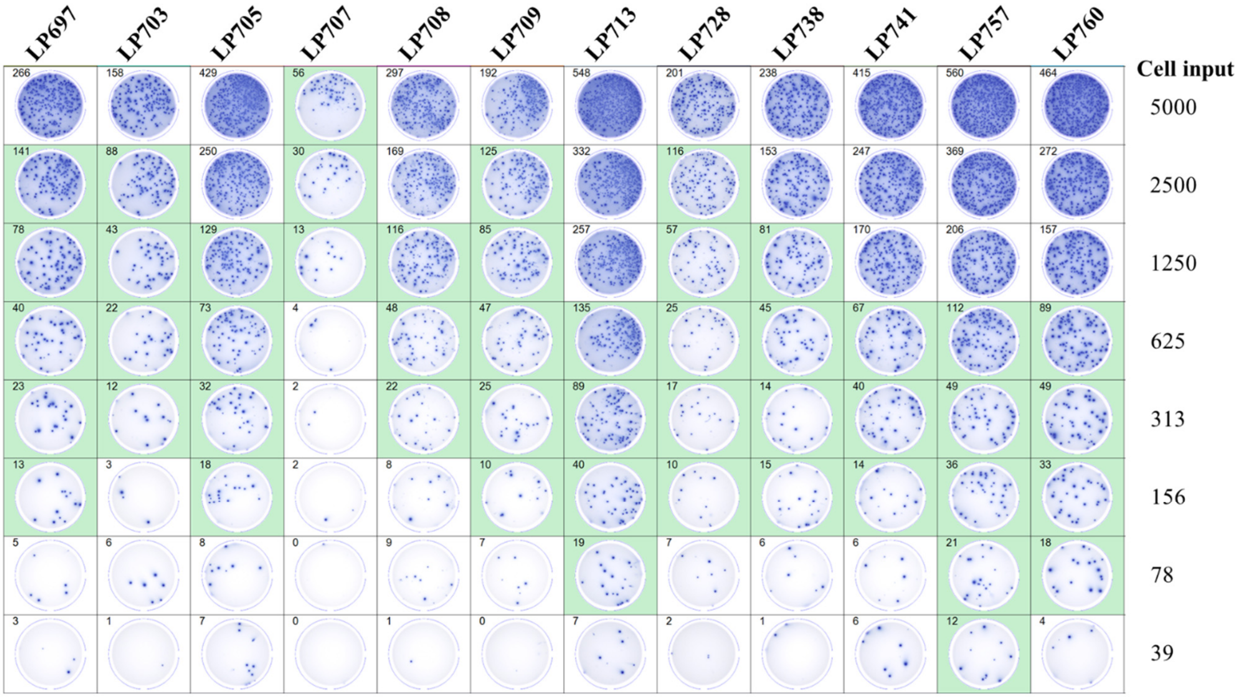

Supplementary Figure S3. Establishing the frequency of pan (total) IgG$^+$ ASC in PBMC following polyclonal stimulation. A representative ELISPOT plate overview depicting the testing of 12 donors (the same as shown in Figure 3) for pan IgG$^+$ ASC activity using a serial dilution approach. The assay principle is illustrated in Suppl. Figure S1A; refer to Section *2.5* for additional details. For each donor the two-fold (1+1) serial dilution was initiated at 5 x 10^3 PBMC/well owing to the much higher frequency of pan IgG$^+$ ASC compared to antigen-specific IgG$^+$ ASC in PBMC following polyclonal stimulation, as specified. The raw images and machine-assisted automated SFU counts are shown in the top left corner for each well. The ImmunoSpot® Studio.SC software (refer to Section *2.5.*) automatically denotes wells yielding SFU counts within a defined upper and lower bound with green shading; for this assay plate 10 to 150 SFU/well. Using contiguous datapoints within the so-called "Goldilocks range" the software automatically calculates the frequency of ASC within all PBMC tested; the autogenerated frequency calculations for these 12 donors are shown in Suppl. Figure S4. Importantly, these data highlight the inter-donor variation in pan IgG$^+$ ASC activity following polyclonal stimulation and the lack of a single cell input concentration in which SFU counts within the Goldilocks range can be obtained for all test subjects.

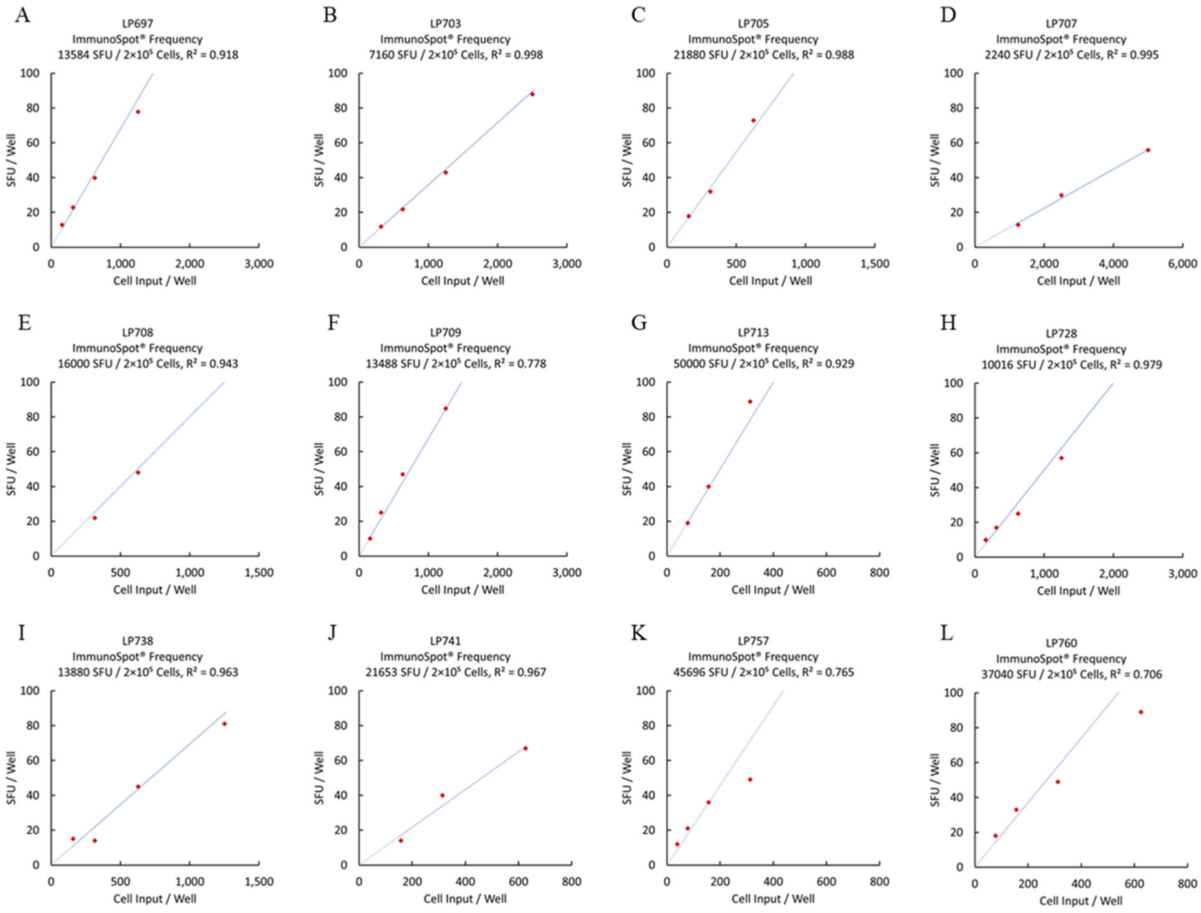

Supplementary Figure S4. Frequency calculations of pan (total) IgG+ ASCs in post-COVID era donors. The automatically generated analysis results by the ImmunoSpot® Studio.SC software are shown for the 12 donors tested in the assay depicted in Suppl. Figure S3 (refer to Section 2.5.). Using 3 or more datapoints within the user-defined "Goldilocks range" (between 10 and 150 SFU/well for this assay plate), the software calculates the frequency of ASC and then extrapolates SFU numbers to a desired cell input (e.g. per 2 x 10^5 PBMC). Additionally, a correlation of determination value, denoted as R^2, is also reported by the software and provides a metric for how closely the raw data at the corresponding cell inputs yielding SFU counts within the linear range fit the calculated frequency; larger R^2 values denote a higher confidence in the reported frequency.

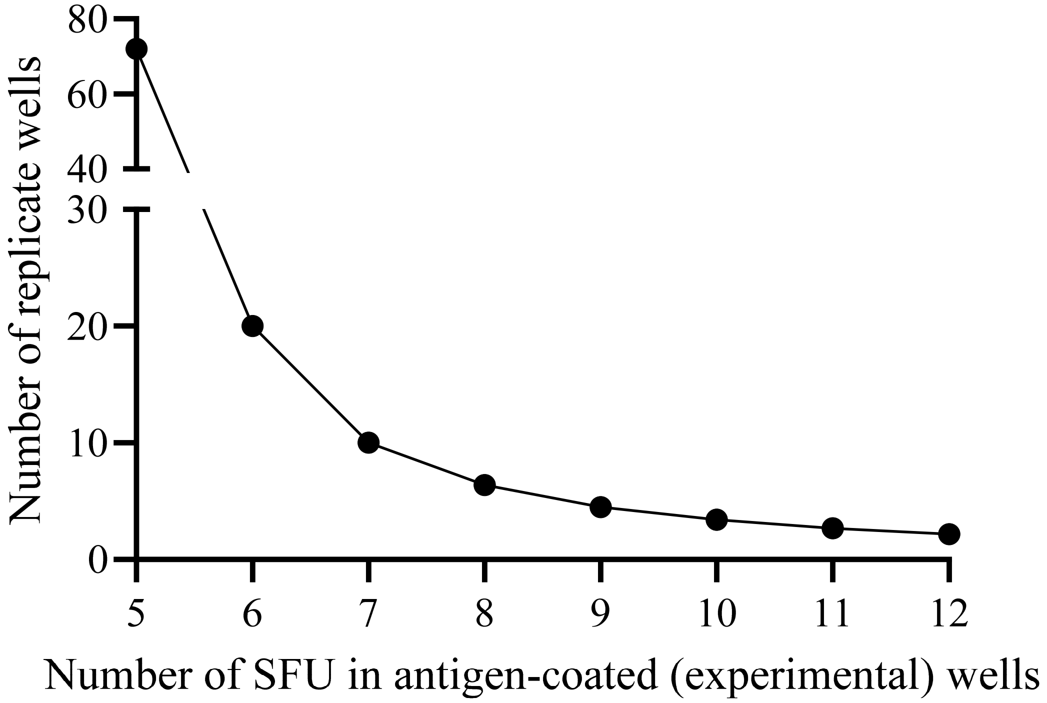

Supplementary Figure S5. Power analysis enables prediction of replicate wells required to measure low frequencies of antigen-specific ASCs with defined level of precision. Assuming a maximal background of 4 SFUs within irrelevant (negative) control wells (see Table 1 in the main text), the minimal number of replicate wells required to detect a positive antigen-specific response in ImmunoSpot assays with power = 90% and significance = 5% is denoted. As shown, the minimal number of replicate wells required is substantially reduced when >10 SFU are detected in antigen-coated (experimental) wells.

Supplementary Table S2. Viral antigen-specific B_{mem}-derived IgG^+ ASC frequencies vary considerably between individual donors. PBMC from healthy individuals (n=20) were tested in ImmunoSpot assays following in vitro polyclonal stimulation to assess B_{mem}-derived IgG^+ ASC reactivity against a panel of recombinantly expressed His-tagged antigens representing ubiquitously encountered viruses. Samples were tested at a fixed cell input of 3 x 10^5 PBMC/well. Note, spot forming unit (SFU) counts that were >100 SFU/well are indicated in bold since quantification of wells with >125 antigen-specific SFUs is generally an underestimate. This Table was reproduced with permission from Becza, *Vaccines*; published by MDPI, 2025 (24).

	Viral Antigens							
Donors	S-antigen (SARS-CoV-2)	NCAP (SARS-CoV-2)	CA/09 rHA (A/H1N1)	TX/12 rHA (A/H3N2)	Phuket/13 rHA (B/Yam)	EBNA1 (EBV)	gH pentamer (HCMV)	6xHis (control)
LP724	**>125**	**>125**	**>125**	4	14	8	1	0
LP726	**>125**	34	94	12	31	21	26	0
LP727	**>125**	**>125**	57	40	34	0	3	0
LP728	85	2	16	18	5	2	0	0
LP730	57	41	50	14	31	14	28	0
LP731	**>125**	5	**>125**	50	**103**	11	0	0
LP735	**>125**	41	6	3	7	4	4	0
LP736	**>125**	14	6	5	13	11	1	0
LP738	**>125**	0	3	15	6	13	17	0
LP739	**>125**	85	29	39	22	**112**	1	0
LP740	**>125**	**118**	19	36	39	79	34	1
LP741	16	1	85	16	26	84	**116**	1
LP749	61	13	6	2	2	29	3	1
LP751	**>125**	**120**	27	28	45	67	23	0
LP756	82	17	21	14	13	28	5	0
LP757	**116**	3	**>125**	11	59	22	0	0
LP758	**>125**	**>125**	77	**>125**	30	36	1	0
LP760	**>125**	19	88	52	50	27	**119**	0
LP761	**>125**	27	**>125**	50	70	**109**	5	1
LP769	**>125**	99	83	42	29	1	4	0

Supplementary Table S3. Increasing the number of PBMC plated per well does not result in a proportional increase in NCAP-specific IgG^+ SFU. PBMC collected from post-COVID era donors (after May 2022, refer to Suppl. Table S1) displaying low frequencies of NCAP-specific IgG^+ SFU in spite of their expected exposure to the SARS-CoV-2 virus were tested both at 2 x 10^5 and 5 x 10^5 PBMC/well in four replicate wells per condition. The expected 2.5-fold increase in SFU counts was not achieved in any of the 12 donors tested. Rather, for many of these donors the secretory footprint morphologies deteriorated and became barely discernable using machine-assisted automated counting (refer to Section 2.5. and raw data in Figure 5).

		NCAP (2 x 10^5) $\bar{x} \pm \sigma$	NCAP (5 x 10^5) $\bar{x} \pm \sigma$	Fold Difference
Post-COVID Era Donors	LP696	10.25 ± 2.86	13 ± 1.22	1.3
	LP701	5.25 ± 2.38	3.25 ± 1.30	0.62
	LP707	5.5 ± 2.18	10.75 ± 2.49	1.95
	LP709	4.75 ± 1.48	7.5 ± 2.87	1.6
	LP741	3.0 ± 1.41	1.25 ± 0.83	0.42
	LP757	10.0 ± 3.24	4.25 ± 2.49	0.43
	LP803	5.25 ± 2.68	1.0 ± 0.71	0.19
	LP820	8.0 ± 1.22	0.5 ± 0.5	0.06
	LP828	6.25 ± 1.79	0.25 ± 0.43	0.04
	LP832	3.75 ± 2.17	1.0 ± 0.71	0.27
	LP834	5.75 ± 1.64	0.25 ± 0.43	0.04
	LP836	4.5 ± 1.50	0.25 ± 0.43	0.06

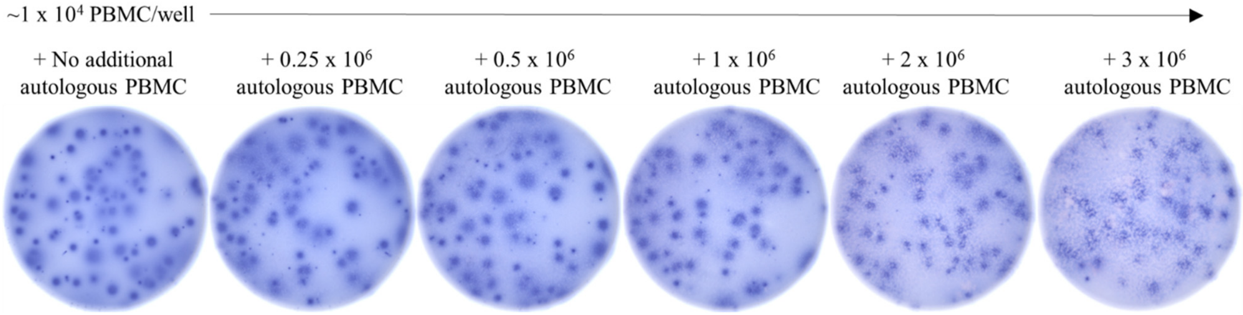

Supplementary Figure S6. Crowding of third-party PBMC interferes with detection of secretory footprints in ImmunoSpot assays. PBMC from a convalescent donor with prior PCR-verified SARS-CoV-2 infection (LP566, refer to Suppl. Table S1) was selected for this experiment owing to their high precursor frequency of S-antigen-specific B_{mem}-derived IgG^+ ASC following polyclonal stimulation. While pristine spot-forming units (SFUs) in the higher Goldilocks range were obtained when their PBMC were plated at 1 x 10^4 cells/well (well on the left), upon adding increasing numbers of freshly thawed (unstimulated) autologous PBMC from the same donor (that therefore contained resting lymphocytes including B_{mem} that were not constitutively secreting IgG) the resulting SFU morphologies began to deteriorate. In contrast to when a total of 2.6 x 10^5 PBMC were plated (containing 1 x 10^4 polyclonally stimulated PBMC and 2.5 x 10^5 unstimulated bystander autologous PBMC) and the resulting SFU morphologies were largely unperturbed (second well image from the left), as the numbers of bystander PBMC increased the ability to detect smaller secretory footprints first became comprised and additionally the larger secretory footprints became more diffuse. Furthermore, when the numbers of bystander PBMC per well exceeded 2 x 10^6 cells the quality of all detectable S-antigen-specific secretory footprints was severely compromised. Collectively, these data clearly demonstrate that crowding of bystander cells at high cell input densities can hinder access of ASC-derived Ig to the assay membrane and undermine the single cell resolution of an ImmunoSpot assay.

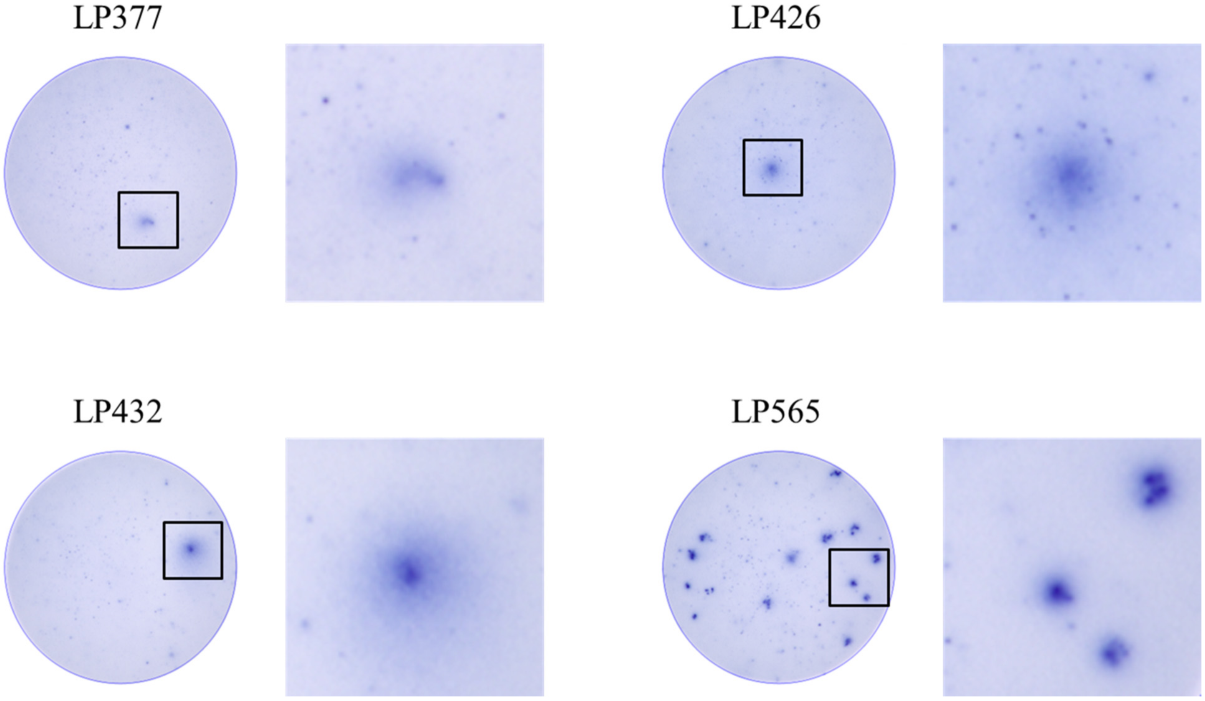

Supplementary Figure S7. Images depicting low-affinity vs. high-affinity NCAP-reactive IgG^+ secretory footprints at higher magnification. Representative well images from an NCAP-specific ImmunoSpot assay (refer to Figure 6) in which PBMC from pre-COVID era (LP377, LP426 or LP432) or a convalescent donor with PCR-verified SARS-CoV-2 infection (LP565) were plated at 2 x 10^5 cells/well are shown. The black box denotes the region of the assay well shown at higher magnification. Note the differential morphology of the secretory footprints generated by the convalescent donor, reflecting the presence of cognate SARS-CoV-2 NCAP-primed B_{mem}, compared to those generated by the pre-COVID era donors that likely originated from low-affinity, cross-reactive B_{mem}.

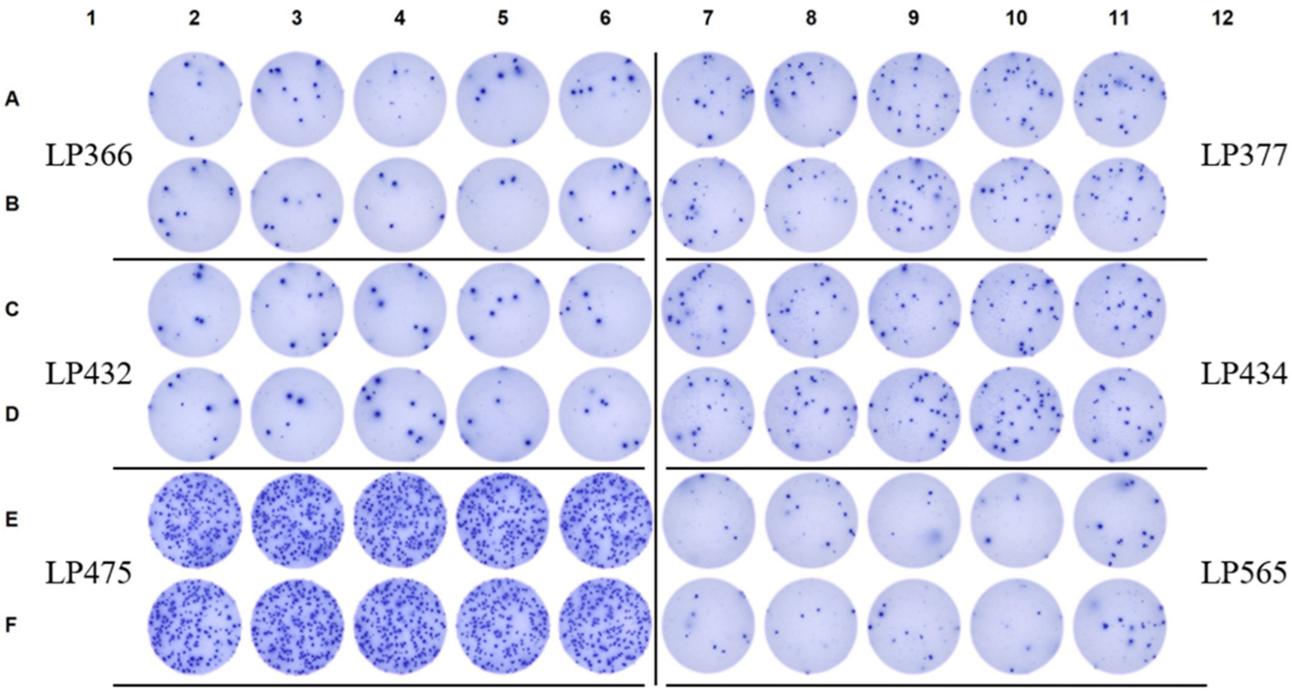

Supplementary Figure S8. High resolution ImmunoSpot testing for influenza (H1)-specific B_{mem}-derived IgG$^+$ SFU in pre-COVID era donors. Raw data are shown from an ImmunoSpot assay in which pre-COVID era donors were seeded at 2 x 10^5 PBMC/well into wells coated with recombinant hemagglutinin (rHA) protein representing a seasonal H1N1 vaccine strain (A/California/2009). Assay specifics were otherwise identical to the data presented in Figure 6 and Suppl. Figure S9. Notably, while frequencies of influenza H1-specific IgG$^+$ SFU were variable among the pre-COVID era donors shown, they were detectable in all subjects and were crisp and dense, i.e., reflective of high-affinity antibody binding. Furthermore, owing to the high abundance of influenza H1-specific B_{mem}-derived IgG$^+$ SFU when LP475 was plated at 2 x 10^5 PBMC/well, a serial dilution approach would be required for an accurate frequency measurement.

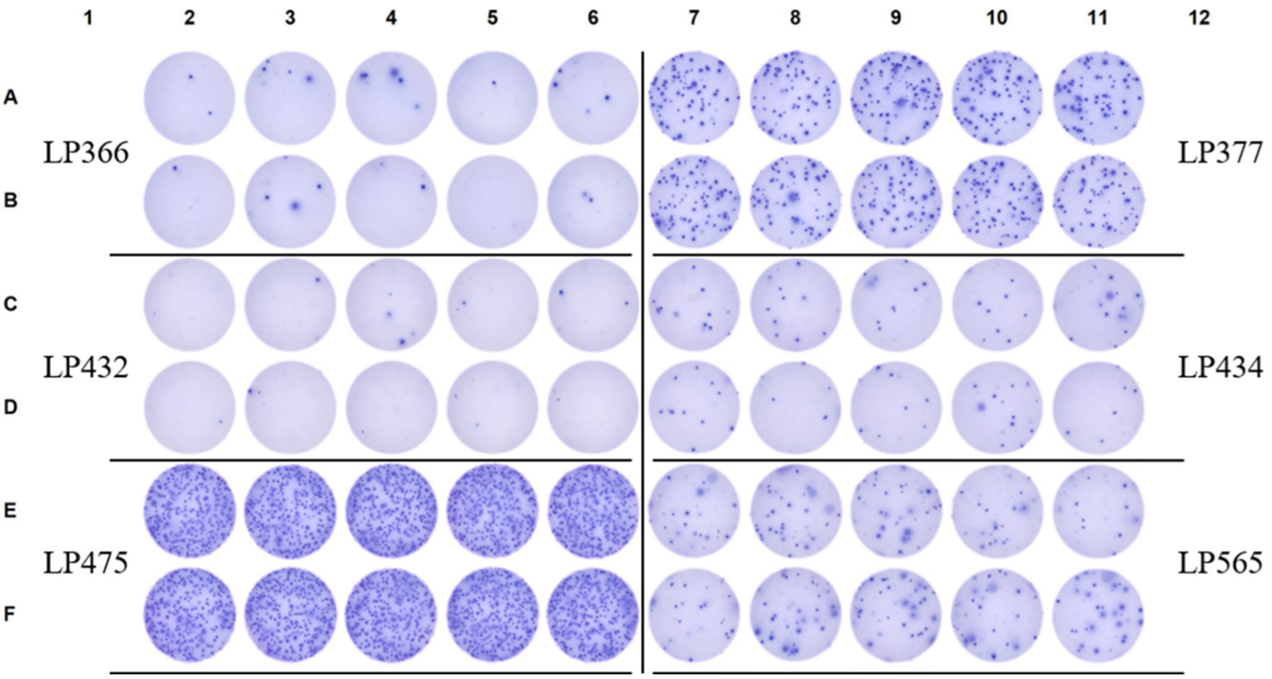

Supplementary Figure S9. High resolution ImmunoSpot testing for influenza B HA-specific B_{mem}-derived IgG^+ SFU in pre-COVID era donors. Raw data are shown from an ImmunoSpot assay in which pre-COVID era donors were seeded at 2×10^5 PBMC/well into wells coated with recombinant hemagglutinin (rHA) protein representing a seasonal influenza B vaccine strain (B/Phuket/2013). Assay specifics were otherwise identical to the data presented in Figure 6 and Suppl. Figure S8. Notably, while frequencies of influenza B HA-specific IgG^+ SFU were variable among the pre-COVID era donors shown, they were detectable in all subjects and were crisp and dense, i.e., reflective of high-affinity antibody binding. Furthermore, owing to the high abundance of influenza B HA-specific B_{mem}-derived IgG^+ SFU when LP475 was plated at 2×10^5 PBMC/well, a serial dilution approach would be required for an accurate frequency measurement.
